# *B*iologic *A*batement and *C*apturing *K*ids’ *O*utcomes and *F*lare *F*requency in *J*uvenile *S*pondylo*a*rthritis (BACK-OFF JSpA): study protocol for a randomized pragmatic trial

**DOI:** 10.1186/s13063-022-07038-6

**Published:** 2023-02-08

**Authors:** Pamela F. Weiss, Cora E. Sears, Timothy G. Brandon, Christopher B. Forrest, Emily Neu, Melanie Kohlheim, Jenny Leal, Rui Xiao, Daniel Lovell

**Affiliations:** 1 Division of Rheumatology and Center for Pediatric Clinical Effectiveness, Roberts Center for Pediatric Research, Children’s Hospital of Philadelphia, 2716 South Street, Room 11121, Philadelphia, PA 19104 USA; 2 Center for Clinical Epidemiology and Biostatistics, Perelman School of Medicine at the University of Pennsylvania, Philadelphia, USA; 3 Applied Clinical Research Center, Children’s Hospital of Philadelphia, Philadelphia, PA USA; 4 Department of Biomedical and Health Informatics, Children’s Hospital of Philadelphia, Philadelphia, PA USA; 5 Department of Pediatrics, Children’s Hospital of Philadelphia, Philadelphia, PA USA; 6 Sidney, OH USA; 7 Granville, OH USA; 8 Columbus, OH USA; 9 Department of Biostatistics, Epidemiology and Informatics, Perelman School of Medicine, University of Pennsylvania, Philadelphia, USA; 10 Department of Pediatrics and Division of Rheumatology at Cincinnati Children’s Hospital Medical Center, University of Cincinnati, Cincinnati, USA

**Keywords:** Spondyloarthritis, Pediatrics, Biologic, Randomized pragmatic trial, Electronic Health Records, Patient-reported outcome

## Abstract

**Background:**

The effectiveness of biologic therapies, primarily tumor necrosis factor inhibitors (TNFi), for children with spondyloarthritis (SpA) has made inactive disease a realistic patient outcome. However, biologic therapies are costly, primarily delivered by subcutaneous or intravenous route, and have non-trivial side effects. Many patients and families want to know if biologic medications can be discontinued after inactive disease is achieved. It remains unclear whether medication dose should remain unchanged, tapered (increase the time between doses), or discontinued once when inactive disease is attained.

**Methods:**

The *B*iologic *A*batement and *C*apturing *K*ids’ *O*utcomes and *F*lare *F*requency in *J*uvenile *SpA* (BACK-OFF JSpA) trial is a multicenter pragmatic trial that will randomize 198 participants ages 8–21 years old with SpA and sustained inactive disease on standard TNFi dosing to (1) continue standard TNFi dosing, (2) fixed longer dosing intervals of TNFi, or (3) stop TNFi. The trial will compare the hazard rate of protocol-defined flare and participants’ emotional health among the 3 groups over 12 months. Innovative aspects of this trial are the involvement of patient and parent stakeholders in the design and conduct of the study as well as an electronic health record-based enhanced recruitment strategy.

**Discussion:**

This is the first randomized pragmatic trial to assess the efficacy of TNFi de-escalation strategies in children with JSpA with sustained inactive disease. This research will improve the evidence base that patients, caregivers, and rheumatologists use to make shared decisions about continued treatment versus de-escalation of TNFi therapy in this population.

**Trial registration:**

ClinicalTrials.gov NCT04891640. Registered on 18 May 2021.

## Administrative information

Note: the numbers in curly brackets in this protocol refer to SPIRIT checklist item numbers. The order of the items has been modified to group similar items (see http://www.equator-network.org/reporting-guidelines/spirit-2013-statement-defining-standard-protocol-items-for-clinical-trials/).

**Table:** 

Title {1}	*B*iologic *A*batement and *C*apturing *K*ids’ *O*utcomes and *F*lare *F*requency in *J*uvenile *S*pondylo*a*rthritis (BACK-OFF JSpA): study protocol for a randomized pragmatic trial.
Trial registration {2a and 2b}.	Clinicaltrials.gov NCT04891640. Registered on 18 May 2021.
Protocol version {3}	Version 3.0; 18 November 2021
Funding {4}	Patient-Centered Outcome Research Institute. National Institutes of Health. Spondylitis Association of America.
Author details {5a}	^1^ Division of Rheumatology, Children’s Hospital of Philadelphia ^2^ Department of Pediatrics and Epidemiology, Perelman School of Medicine, University of Pennsylvania ^3^ Division of General Pediatrics, Children’s Hospital of Philadelphia ^4^ Parent stakeholders and trial co-investigators ^5^ Department of Biostatistics, Epidemiology and Informatics, Perelman School of Medicine, University of Pennsylvania ^6^ Department of Pediatrics, Division of Pediatric Rheumatology, Cincinnati Children’s Hospital Medical Center
Name and contact information for the trial sponsor {5b}	Pamela Weiss, MD, MSCE, Children’s Hospital of Philadelphia, Philadelphia, PA.
Role of sponsor {5c}	Pamela Weiss is the Principal Investigator for BACK-OFF JSpA and was responsible for the trial design, drafting of this manuscript, and the decision to submit the trial protocol for publication. This study was funded through a Patient Centered Outcomes Research Institute® (PCORI®) Award (CER-2020C1-19212). The study design, analysis, and interpretation of results presented in this publication are solely the responsibility of the authors and do not necessarily represent the views of the Patient-Centered Outcomes Research Institute® (PCORI®), its Board of Governors or Methodology Committee.

## Introduction

### Background and rationale {6a}

Approximately 300,000 children in the US are estimated to have arthritis, 10–30% of whom have spondyloarthritis (SpA) [1–4]. SpA is characterized by inflammatory arthritis, enthesitis (tender tendon insertions), dactylitis (swollen fingers), back pain, inflammatory bowel disease, eye inflammation, and psoriasis. Since the introduction of biologic disease-modifying agents such as TNFi, inactive disease has become a realistic goal. In 2018 an international task force of pediatric rheumatologists developed recommendations for treating juvenile arthritis to target [5]. The primary treatment target for juvenile arthritis was inactive disease, defined as the absence of all clinical signs and patient-experienced symptoms of inflammatory disease activity. Current treatment approaches for children with SpA have resulted in up to 60% attaining inactive disease while on therapy [6–8]. Additionally, the international pediatric task force specified several overarching principles for the management of juvenile arthritis which included not only controlling signs and symptoms of disease but also avoidance of drug toxicities and optimization of personal well-being.

The effectiveness of biologic therapies for patients with SpA has made inactive disease a feasible target. However, biologic therapies are costly, primarily delivered by subcutaneous or intravenous route, and have non-trivial side effects. In a report from 2016, the annual direct cost of TNFi per treated adult ranged from $24,859 to $26,537 across indications [9]. Of the 5 TNFis being used to treat children in everyday practice (etanercept, adalimumab, infliximab, certolizumab, golimumab), all are administered subcutaneously except infliximab which is given by intravenous infusion. The use of injectable biologics can also impact personal well-being, anxiety, and sense of “being different” in children [10]. Biologics are associated with increased risk of infection, injection site reaction and pain, psoriasis, demyelinating disorders, and autoantibody development [6, 7, 11]. The association of biologics with risk of malignancy, specifically lymphoma, remains controversial and there is a FDA box warning regarding this risk in the pediatric population [12–18].

Therefore, many patients and families want to know if inactive disease can be maintained if biologics are de-escalated or discontinued. However, there is no evidence to inform whether tapering (increasing the time between doses) or stopping TNFi is advisable after the disease is quiet. Furthermore, if TNFi therapy is de-escalated, it is unclear if tapering or stopping is better. Two small studies of children with all types of juvenile arthritis suggest medication tapering (versus abrupt stopping) is associated with lower rates of flare [19, 20]. A survey of North American pediatric rheumatologists demonstrated significant heterogeneity in TNFi de-escalation strategies for juvenile arthritis [21]. The most commonly used strategies included tapering for several months and then stopping, maintenance of fixed longer intervals, and stopping immediately. All of the TNFi strategies being compared in this trial are currently in use at sites across the USA and there is a strong desire by physicians, patients, and families to understand the best approach. Furthermore, there is no information available to address the effect of therapy de-escalation on patient and caregiver’s lived experiences.

The BACK-OFF JSpA trial is the first clinical trial to examine the safety and efficacy of de-escalating TNFi therapy in children with SpA and sustained inactive disease on treatment and will greatly improve the evidence base that patients, caregivers, and rheumatologists use to make shared decisions about therapy. Innovative aspects of this trial include involvement of patient and caregiver stakeholders in the design and conduct of the study and an electronic health record-based enhanced recruitment strategy. Additionally, the trial will also collect clinical data to assess rates and ease of re-establishing inactive disease after flare. Furthermore, ancillary studies will collect serologic and imaging data to assess predictors of flare and rates of anti-TNFi antibody development. Embedding this research in real-world clinical settings ensures our results are directly translatable to usual patient care and highly impactful.

### Objectives {7}

This trial has two primary objectives:

1. To compare the likelihood of disease flare associated with fixed standard dosing, fixed longer dosing intervals, or stopping tumor necrosis factor inhibitor (TNFi) in children with SpA who have inactive disease. *The primary hypothesis is the hazard rate of children who flare on the fixed longer dosing interval arm will not be inferior to those who stay on fixed standard dosing. The secondary hypothesis is children on the stopping arm will have a higher hazard rate of flare than the other 2 arms.*
2. To compare patients’ lived experiences amongst the three treatment arms. Using the validated Patient-Reported Outcomes Measurement Information System (PROMIS) pediatric pain interference measure, we will assess the children’s self-assessments of their emotional health in the three treatment arms. *The hypothesis is that PROMIS pain interference T-scores from patients randomized to the fixed longer dosing interval arm or stopping arm will be lower over time compared to the fixed standard dosing arm.*

### Trial design {8}

BACK-OFF JSpA is a prospective, 12-month pragmatic randomized trial embedded within routine clinical care. Children with SpA who have maintained inactive disease on standard dosing of a TNFi for 6 months or longer will be eligible for enrollment. Treatment with standard dosing of any of the following TNFis as part of clinical care is acceptable: adalimumab, certolizumab, etanercept, golimumab, infliximab (Table 1).

**Table 1:** Standard dosing and frequency reference

TNFi medication	Standard dose (mg)	Standard dose frequency (weeks)
Adalimumab	40 mg (if ≥30 kg) subq	Every 2 weeks
	20 mg (if <30 kg) subq	
Certolizumab	50 mg (if <40 kg) subq	Every 2 weeks
	100 mg (if ≥40 kg) subq	
Etanercept	0.8 mg/kg/dose, max 50 kg subq	Weekly
Golimumab	80 mg/m^2^ IV	Every 8 weeks (IV)
	30 mg/m^2^, max 50 mg subq	Every 4 weeks (subq)
Infliximab	5–10 mg/kg/dose IV	Every 4–8 weeks

## Methods: participants, interventions, and outcomes

### Study setting {9}

Twenty-one sites across the USA will participate in the trial. All sites have established expertise in juvenile arthritis research and a track record of successful recruitment for prior clinical trials. Nine of the centers are members of PEDSnet, a pediatric clinical research network, that will be leveraged for the enhanced recruitment and conduct of the trial. PEDSnet is one of 8 clinical research networks that constitute PCORnet®, the National Patient-Centered Clinical Research Network [22]. Three additional participating centers are members of PCORnet. PCORnet seeks to improve the nation’s capacity to conduct clinical research by bringing together these clinical research networks to create a large, highly representative, national patient-centered network that supports more efficient clinical trials and observational studies.

### Who will take informed consent? {26a}

The BACK-OFF JSpA Research Partners Group, which consists of caregiver and patient stakeholders, developed study information/recruitment materials for distribution to the family prior to the visit by the local rheumatology team or at the visit. The local team will review the risk and benefits of the trial and answer families’ questions. The treating clinician or clinical research coordinator (CRC) will obtain written or electronic informed consent of caregivers or patients who are 18 years or older and assent of patients less than 18 years old.

### Eligibility criteria {10}

- Age 8–21 years and symptom onset prior to age 16 (ERA criteria) or 18 years (PRINTO criteria) depending on diagnosis criteria met
- Fulfill International League of Associations for Rheumatology criteria for juvenile idiopathic arthritis enthesitis-related arthritis subtype [23] (pediatric form of SpA) or the new provisional Pediatric Rheumatology International Trials Organization (PRINTO) enthesitis-related arthritis criteria [24]
- Current therapy with a TNFi at standard dosing intervals (Table 1)
- Clinically inactive disease for *at least* 6 months, as determined by treating physician
- Interested in and willing to de-escalate TNFi therapy

Patients are eligible for inclusion regardless of the number of joints previously affected and whether there is coexistent axial arthritis. Patients who are receiving concurrent conventional synthetic disease-modifying antirheumatic drug therapy will also be eligible for enrollment.

Any of the following will exclude a participant from enrollment as these conditions may influence the treatment decision independent of the arthritis:

- History of inflammatory bowel disease
- History of psoriasis that pre-dates the start of TNFi therapy or psoriasis that started after TNFi therapy and has required more than topical therapy for control
- History of uveitis

### Additional consent provisions for collection and use of participant data and biological specimens {26b}

Contribution of serologic samples and imaging data is optional and funding for these procedures is distinct from the BACK-OFF JSpA trial. Participants who decline serologic samples or imaging may still enroll in the trial. All children who consent to provide serologic samples will have a peripheral blood draw within 4 weeks of randomization and again at the time of flare or at the end of the trial (12 months post-randomization) if no flare. Children with a history of axial disease who consent to imaging will undergo a dedicated pelvic magnetic resonance imaging (MRI) within 4 weeks of randomization and again at the time of flare or at the end of the trial (12 months post-randomization) if no flare.

## Interventions

### Explanation for the choice of comparators {6b}

The three alternative approaches being compared are (1) fixed standard dosing (i.e., no change from current therapy), (2) fixed longer dosing intervals of TNFi (i.e., increased time between doses), and (3) stopping TNFi*.* Pediatric rheumatologists in North America are variable in their TNFi de-escalation strategies due to lack of evidence on the topic. Clinicians at the participating centers agreed there is clinical equipoise with respect to these approaches.

### Intervention description {11a}

**Fig. 1:**
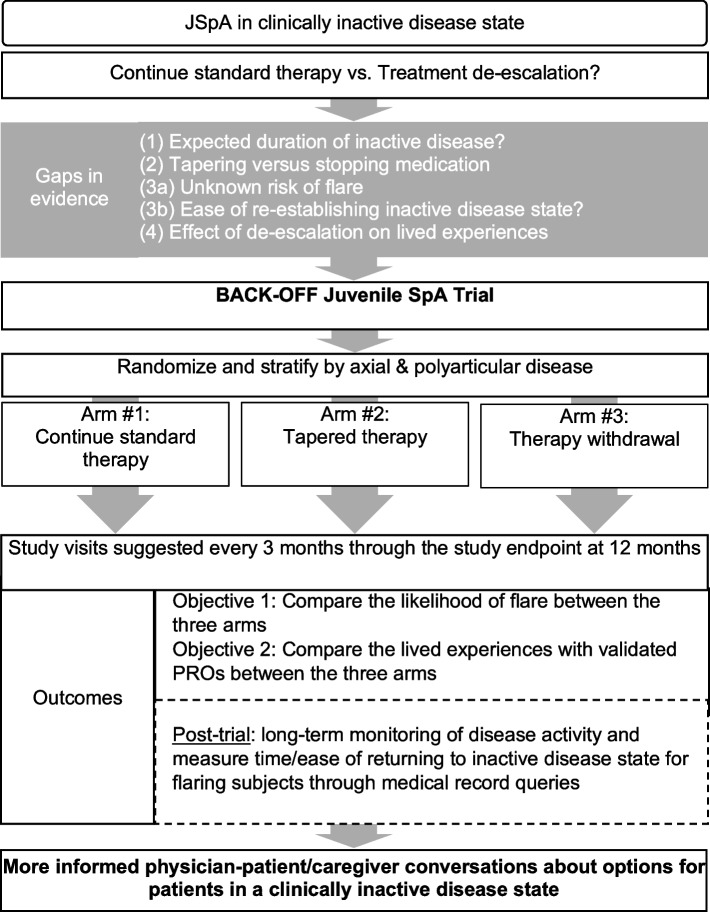
Conceptual framework for BACK-OFF JSpA Trial

One hundred and ninety-eight children will be randomized to continued fixed standard dosing (arm 1), fixed longer dosing intervals of TNFi (arm 2), or stopping TNFi (arm 3) (Fig. 1). Details of TNFi dose interval prolongation are in the legend of Fig. 1. The duration of the intervention period will be 12 months or until a flare occurs, whichever happens first.

### Criteria for discontinuing or modifying allocated interventions {11b}

Participants who experience a fever of at least 101 F or an infection requiring antibiotics should consult their clinician regarding treatment; if deemed necessary, participants may hold or stop their medication as would be done as part of routine care. Participants should resume their treatment once cleared by their clinicians to do so. Additionally, participants who experience a serious adverse event, adverse event that impacts systemic therapy, or a pregnancy should consult their clinician regarding treatment and hold or stop their medication as would be done as part of routine care.

### Strategies to improve adherence to interventions {11c}

Subject adherence to intervention assignment will be assessed through patient/caregiver self-report as is typical for routine care. Subjects will be encouraged to follow their assigned intervention’s medication schedule as closely as possible.

### Relevant concomitant care permitted or prohibited during the trial {11d}

Patients who are receiving conventional synthetic disease-modifying antirheumatic drug (DMARD) therapy along with standard dosing of a TNFi are eligible for enrollment. The dose of the DMARD can be changed during the study as per the treating clinician and family preference; this will be accounted for in the analysis. Topical, inhaled, or ophthalmic steroids are permitted as needed. Oral NSAIDs are permitted as per routine care. Intraarticular corticosteroids are not permitted prior to protocol-defined flare.

### Provisions for post-trial care {30}

If a patient meets protocol-defined flare, then he/she would no longer be part of the active part of the trial, and it would be up to the patient and their local rheumatologist to choose which medicine to use going forward. If a patient suffers an adverse event, then subsequent care is delineated as per the treating physician and standard clinical care practices.

### Outcomes {12}

For the first objective, the primary outcome is disease flare, which is defined as a clinically meaningful worsening in 3 or more core variables, using the visit at time of randomization as the reference (Table 2) [25]. The flare definition for this study was designed to mimic the clinical decision made at the point-of-care to reinitiate or not reinitiate TNFi therapy, validated in an open-label situation, and demonstrated excellent measurement characteristics [25].

**Table 2:** Protocol-defined flare [25]

Flare core variable	Range of scores	Clinically meaningful change
1. Caregiver/patient assessment of overall well-being VAS	0–10 (0=maximal well-being)	≥2
2. Physician assessment of disease activity VAS	0–10 (0=no disease activity)	≥2
3. Caregiver/patient assessment of pain VAS	0–10 (0=no pain)	≥2
4. Function (PROMIS mobility and upper extremity)	Mean *T*-score=50 (SD 10)	≥3
5. Active joint count	0–10	≥1

### Participant timeline {13}

After participants are consented, eligibility confirmed, and randomization assignment given, they will have routine clinic visits every 3 months until flare or the end of the intervention period. Additional unscheduled visits will occur as needed for concern for flare. Study procedures and timeline are shown in Table 3.

**Table 3:**
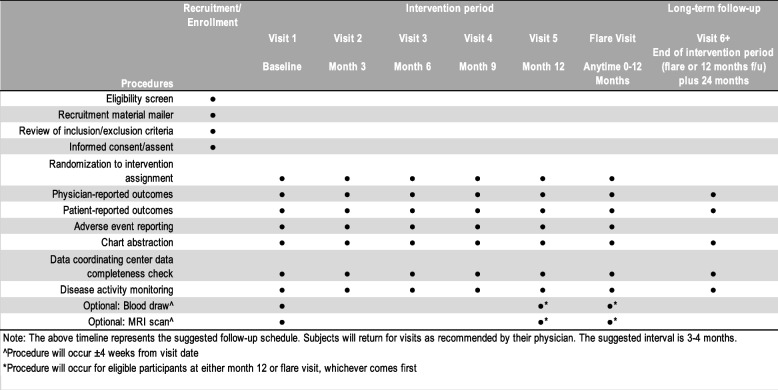
Study procedures and visit timeline

### Sample size {14}

We aim to recruit 198 subjects in total with 66 randomized to each arm, with an anticipated 10% loss to follow-up. Based on the data in the C-OPTIMISE trial [26], we anticipate the cumulative flare rate in the BACK-OFF JSpA standard dosing group by month 12 (end of the active study) will be approximately 16%. With 60 patients in standard treatment group and 60 in fixed longer dosing group, and a 16% cumulative flare rate in the standard dosing group, a non-inferiority comparison using the logrank test achieves 80% power at a 0.05 significance level when the non-inferiority bound is 2.5 and the hazard ratio (HR) is 1. Stated simply, if the cumulative rate of flare in the standard TNFi dose group is 16%,

we can declare noninferiority if the HR non-inferiority bound is ≤2.5 and the cumulative flare rate in the fixed longer dosing arm is no more than 31%. This meets the acceptable noninferiority margin of an absolute difference of 15% in the cumulative flare rate that was determined by consensus of BACK-OFF JSpA site PIs. This non-inferiority margin is also in accordance with other recent published TNFi dose reduction trials [27–30]. To test our secondary hypothesis, which is that the TNFi stop arm is inferior to each of other two arms (standard TNFi dose and fixed longer TNFi dosing), the logrank test achieves 80% power at a 0.025 significance level (to adjust for multiple comparisons of the secondary hypotheses) to detect a difference of 3.2 in the hazard ratio*.*

For Objective 2, with an evaluable *N*=60 in each arm we will have 92% power at a significance level of 0.02 to detect a time-average difference of 0.5×SD between any two arms in pain interference if we account for repeated measures (3 times on average, ranging from 2 to 4) by assuming a within-subject correlation of 0.3. FDA guidance suggests that 0.5 standard deviation (SD) units is the level of group-level change in PROs that is clinically meaningful, which we will be powered to detect [31].

### Recruitment {15}

The foundation of our recruitment strategy for the BACK-OFF JSpA trial is based on preferences stated by participants of our family study design studio and incorporates both traditional clinic-screening-based approaches as well as an enhanced population-based strategy that leverages the electronic health record (EHR). Through the use of an EHR-enhanced recruitment approach, we aim to reduce the screening efforts required to identify eligible patients, enable the team to introduce families to the trial, and share patient- and parent-generated recruitment materials 1–2 weeks prior to upcoming visits in rheumatology, and reduce the burden on on-site teams to provide recruitment materials and study information at point-of-care. PEDSnet (pedsnet.org) is a multi-institutional clinical research network that aggregates EHR data from approximately half of the BACK-OFF JSpA sites. We constructed an EHR-based screening algorithm, optimized for sensitivity, to identify patients with SpA followed in rheumatology clinic. The algorithm was 99% accurate in rejecting patients with a very low chance of meeting eligibility (i.e., non-SpA patients), thereby providing reassurance that the gateway code is specific. Using chart review as the reference standard, after application of inclusion and exclusion criteria to SpA patients who met the gateway code, the screening algorithm had sensitivity of 93% and a PPV of 93%, in identification of eligible patients. For non-PEDSnet sites, the typology is based upon that developed for PEDSnet sites but adapted to the local EHR platform.

**Fig. 2:**
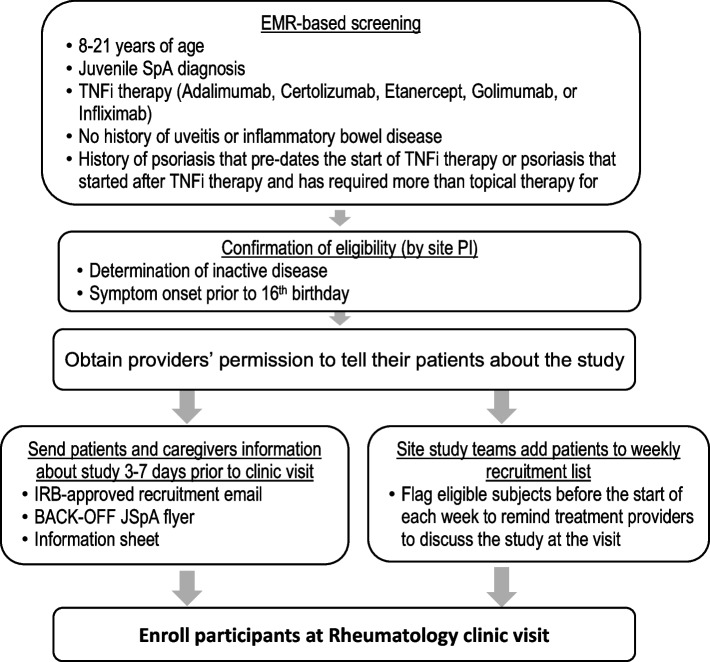
Recruitment plan

Once potential subjects are identified through the central EHR query, families with trial eligibility confirmed by site study staff will receive recruitment materials within 1–2 weeks of their upcoming rheumatology visit. Each site will generate weekly lists of eligible patients based on the queries as described above plus a review of scheduled visits for the coming week (Fig. 2).

## Assignment of interventions: allocation

### Sequence generation {16a}

Randomization will be 1:1:1 and providers and patients will be unblinded due to the need to measure the lived experiences in each study arm. Certain patient characteristics, such as the number of joints ever involved (<4 or ≥5) and presence of axial disease, may confound or influence the primary outcomes of flare (Aim 1) and patient-reported measures (Aim 2). To ensure a balanced distribution of participants with these characteristics across the 3 arms, enrolled participants will be randomly assigned to an arm stratified by oligo- versus polyarticular disease and axial disease (i.e., 4 strata in total: oligo without axial disease, oligo with axial disease, polyarticular without axial disease, and polyarticular with axial disease).

### Concealment mechanism {16b}

Randomization will take place remotely via web-based assignment within the Research Electronic Data Capture (REDCap), applying the criteria stated above. The coordinating center will upload the allocation sequence generated by the study biostatistician into REDCap. The users at each site will be blinded to the randomization sequence.

### Implementation {16c}

After the local research team has consented and enrolled a subject, the local treatment assigner will verify the subject ID, strata assignment, and the date of randomization. REDCap will subsequently generate the subject’s randomization assignment.

## Assignment of interventions: Blinding

### Who will be blinded {17a}

Treatment assignment is unblinded

### Procedure for unblinding if needed {17b}

Not applicable.

## Data collection and management

### Plans for assessment and collection of outcomes {18a}

The primary outcome of protocol-defined flare will be assessed real-time at each study visit. Additional information regarding patient-reported outcomes, medications, adherence, and adverse events will be collected at each study visit on tablets directly from the subjects using the REDCap Mobile Application.

### Plans to promote participant retention and complete follow-up {18b}

Given the eligibility criteria, the majority of participants will be under the long-term care of the enrolling investigator. Additionally, the following efforts exist to promote retention: stipends to each participant for each study visit to reimburse for time; reminder emails and telephone calls about upcoming visits from the data coordinating center to the site study coordinator; reminder emails and telephone calls about upcoming visits from the site study coordinator to the caregivers and/or patient if over 18 years. Each site’s study coordinator will collect at least 2 contact numbers of caregivers and from the patients if over 18 years. All contact information will be verified at each visit. The BACK-OFF JSpA Research Partners group will utilize social media accounts to post study updates and news relevant to families with SpA.

### Data management {19}

Data will be entered directly into the REDCap database using encrypted tablets requiring login credentials for the tablet and for the REDCap Mobile App. REDCap is programmed with validation requirements to prevent entry of out-of-range data. Completeness of research data and determination of protocol-defined “flare” versus “no flare” will be determined by the data coordinating center at the time of the study visit; flare is based upon clinically meaningful change compared to the index visit in ≥3 of 5 core measures [25]. Additionally, verification of data entry completion will occur in real time during the visit via direct review of data forms at the coordinating center and iterative interaction with the site. If a participant isn’t able to complete all items during a study visit (if not related to the primary outcome), they will be contacted with a request to complete their questionnaires through a secure web link generated by REDCap with reminders several days later.

REDCap is a secure, web-based software toolset and workflow methodology for electronic collection and management of research data, developed specifically around HIPAA-Security guidelines. REDCap provides easy data manipulation, including audit trails for reporting, monitoring, and querying patient records. The REDCap MySQL database is replicated in real-time to a completely redundant instance of MySQL. The redundant instance is available for restoration of the primary database or for manual failover in the case of primary database failure. Time-stamped backup files are made from the replicated database daily by CHOP Information Systems using automated backup routines.

Data and backups are stored in the CHOP Information Systems Storage Area Network (SAN). Access to the SAN directories where data are stored will be limited to Information Systems personnel, with authentication performed using CHOP’s enterprise Active Directory service.

### Confidentiality {27}

The IRB-approved HIPPA waiver of authorization allows for screening procedures to be practicably performed without formal written consent from patients beforehand. Participants who consent and enroll are assigned a unique, randomly generated ID to protect their privacy. Only the local institution and CHOP data team members will have access to Patient Health Information.

### Plans for collection, laboratory evaluation and storage of biological specimens for genetic or molecular analysis in this trial/future use {33}

Subjects can opt-in to participate in ancillary studies and have serum drawn for cellular biomarkers, TNFi antibody and drug levels, and biobanking, preferably coupled with standard laboratory blood draws. Serologic samples will be processed locally, frozen, and batch shipped to the laboratory of Dr. Salvatore Albani (Duke NUS Medical School) in Singapore for the analysis of cellular biomarkers of flare. Serologic samples will also be shipped to the laboratory of Dr. Rae Yeung, PI of the UCAN-CAN DU Registry (Toronto, Ontario) for exploratory cytokine and mRNA expression analyses. The TNFi antibody and drug level samples will be frozen and batch shipped to the Exeter Clinical Laboratory at the Royal Devon and Exeter Hospital (United Kingdom).

## Statistical methods

### Statistical methods for primary and secondary outcomes {20a}

For Objective 1 the primary analysis will be intention-to-treat (ITT). We will use Cox proportional hazard regression for the non-inferiority hypothesis assessing the hazard ratio between the fixed longer dosing interval group and those in the continued standard therapy group. We then compare the HR between the arms to the pre-specified non-inferiority margin of clinical interest. However, for non-inferiority trials, it is known that ITT analysis tends to bias towards the null, which may lead to false claims of non-inferiority [32]. As such, we will also perform a per-protocol analysis and report the results from both**.** For Objective 1 hypothesis 2, we will compare the HR of flare in children who are randomized to stop therapy (Arm 3) with those randomized to Arms 1 or 2 using cox proportional hazard regression and ITT analysis, with the ‘therapy withdrawal’ group as the reference group.

For Objective 2, comparing patients’ patient-reported outcomes amongst the 3 arms, the primary outcome is pain interference and we will use ITT analysis. We will compare the unadjusted mean in the pain interference T-scores between the three intervention arms at each visit, and also use linear mixed-effects regression for repeated measures to compare the change in *T*-scores over time between the intervention arms. This approach also allows us to model the individual-level trajectories by including subject-specific intercept and slope.

### Interim analyses {21b}

There are no planned interim analyses.

### Methods for additional analyses (e.g. subgroup analyses) {20b}

We plan to assess for heterogeneity of treatment effect amongst those with polyarticular or axial involvement in exploratory analyses.

### Methods in analysis to handle protocol non-adherence and any statistical methods to handle missing data {20c}

The primary analysis for both trial objectives will be ITT where participants will be included based on their randomization assignment regardless of adherence. Subjects who report non-adherence and do not plan to adhere going forward will be censored after the visit at which they confirm non-adherence. Subjects who are lost to follow-up will be included in the analysis; their time will be censored after their last study visit. A secondary per-protocol analysis will also be conducted as a sensitivity analysis.

### Plans to give access to the full protocol, participant-level data and statistical code {31c}

BACK-OFF JSpA is registered on ClinicalTrials.gov (NCT04891640). De-identified participant-level data and statistical code from the trial may be provided by request to researchers who provide a methodologically sound proposal or for participant data meta-analysis at the discretion of the BACK-OFF steering committee. To gain access, data requestors will need to sign a data access agreement.

## Oversight and monitoring

### Composition of the coordinating center and trial steering committee {5d}

The BACK-OFF JSpA Coordinating Center is composed of clinician-scientists, biostatisticians, and master-level researchers to provide oversight and implementation of the trial at all study sites. Responsibilities of the Coordinating Center include maintaining up-to-date human research approvals, conducting training and certification of local research teams, supervising the trial activities at all sites, and monitoring of recruitment, protocol adherence, data quality and completeness, and safety. The trial steering committee is composed of the study Principal Investigator, 2 clinical scientist co-investigators, a biostatistician, and 3 parent-partner co-investigators. The BACK-OFF JSpA Research Partners Group includes patients with SpA who are treated with TNFi, caregivers from participating sites, representatives from national SpA-related advocacy and education organizations, rheumatologists, and payer representatives.

### Composition of the data monitoring committee, its role and reporting structure {21a}

An independent Data Safety Monitoring Committee was formed consisting of pediatric rheumatologists and biostatisticians. The DSMC will meet approximately every 6 months to evaluate the study progress and any grade 3 or higher adverse events.

### Adverse event reporting and harms {22}

All subjects will be asked about adverse events at every visit. Adverse events determined to be grade 3 or higher according to the Common Terminology Criteria for Adverse Events v5.0 (CTCAE) will be documented throughout the study. If a serious adverse event occurs, the site should report the event to the coordinating center within 48 h of knowledge of the event. BACK-OFF JSpA trial will also collect new or worsening of existing autoimmune conditions as event(s) of special interest.

### Frequency and plans for auditing trial conduct {23}

The BACK-OFF JSpA Coordinating Center will evaluate the quality of trial activities on at least a monthly basis, more often at study launch. Weekly communication will be shared with the research group regarding trial progress and protocol adherence.

### Plans for communicating important protocol amendments to relevant parties (e.g. trial participants, ethical committees) {25}

All protocol amendments must be approved by the PCORI oversight committee. Following PCORI’s approval, amendments will be submitted to the CHOP IRB, which is serving as the reviewing IRB for this multi-center study. Once approved by the CHOP IRB, collaborating sites will be informed of protocol amendments via email and all amended protocol documents will be uploaded into the protected sharefile platform, which all collaborating site personnel can access. Each local site is responsible for submitting amendment changes to their local IRBs.

### Dissemination plans {31a}

Clinicians and the BACK-OFF JSpA Research Partners Group will partner in the interpretation of the results before any reports are disseminated. We will develop guides detailing patient and caregiver experiences with TNFi de-escalation. We will further enhance dissemination through partnership with SpA Association of America, Arthritis National Research Foundation, and our payer stakeholders, each of which has websites and ways to disseminate research findings to their membership including email blasts, social media, and webinars. Study results will be presented to the physician community (pediatric and adult rheumatologists) at national meetings, published in research journals, and highlighted in publications that summarize studies of importance.

## Discussion

The *B*iologic *A*batement and *C*apturing *K*ids’ *O*utcomes and *F*lare *F*requency in *J*uvenile *SpA* (BACK-OFF JSpA) is the first study of its kind to assess the efficacy of TNFi de-escalation strategies in children with SpA with sustained inactive disease. Goals of evaluating de-escalation strategies are to reduce unnecessary exposure to costly immunosuppressants that have non-trial side effects and to reduce patients’ symptom burden. The trial is open-label and conducted in the context of routine clinical care. The novelty of this trial is evidenced not only by the rare disease being studied, the context of the study, and the questions being asked, but also by the involvement of patient and caregiver stakeholders and the enhanced recruitment strategy.

Juvenile SpA patients and their caregivers played a key role in the design of this study. In preparation for the study, we conducted a family study design studio (focus group) to gain insight into the trial design preferences of patients and parents within our study population to provide them with the opportunity to offer suggestions and voice concerns based on their own experiences. The decision to make the study a 3-arm trial inclusive of a continuation of standard therapy arm resulted from this studio. From the studio, we also learned about preferences for receipt of recruitment materials and how these differed for patients and their caregivers. Their stated preferences formed the basis of our recruitment strategy. In addition, our stakeholders participated in a discrete choice experiment to evaluate their preferences regarding patient-reported outcomes that should be prioritized for the BACK-OFF JSpA trial [33]. This exercise resulted in an estimate of the relative importance and rank order of 21 NIH pediatric PROMIS pediatric outcome domains. The rank-order list directly informed the primary and secondary patient-reported outcomes for Aim 2 of the trial. Since patient-reported outcomes are included in the updated OMERACT JIA core set [5], stakeholder input into the design and/or conduct of all JIA trials should be contemplated. As it relates to the BACK-OFF JSpA trial, if risk of disease flare is non-inferior between the continue and fixed longer dosing interval strategies being evaluated, differences in patient-reported outcomes will be tremendously informative helping patients and caregivers decide which strategy is right for them.

In addition to clarifying preferences for timing, method of distribution, and content of recruitment information our patient and caregiver stakeholders designed the BACK-OFF JSpA recruitment flyers and made a video. Given that recruitment and enrollment is one of the top reasons for trial delay and failure we felt that hearing from patients and caregivers about why eligible participants should want to participate would be quite powerful. In addition, sending these materials to potential participants in a timely fashion before an upcoming rheumatology evaluation was felt to be key. To facilitate this effort we partnered with PEDSnet, a national pediatric clinical research network, to develop an EHR population-based approach to enhance each site’s ability to efficiently and systematically identify eligible patients with JSpA. Through the EHR query, we can ascertain which patients have upcoming rheumatology visits and can pre-inform patients about trial eligibility. This population-based EMR-screening approach will reduce not only the burden of local team screening efforts but also the burden on-site teams to provide recruitment materials and study information at point-of-care. Importantly, this advance notice also gives families valuable time to contemplate participation and ample time to formulate questions for the study team.

In summary, BACK-OFF JSpA is a randomized pragmatic trial to assess the efficacy of TNFi de-escalation strategies in JSpA with sustained inactive disease. The unblinded and open-label approach will enable the evaluation of the patients’ lived experiences and result in an approximation of the effect in routine clinical care. The successful and novel involvement of key stakeholders will hopefully inspire other trials to similarly weave patients and caregivers into study design and conduct. This research will greatly improve the evidence base that patients, caregivers, and rheumatologists use to make shared decisions about continued treatment versus de-escalation of TNFi therapy in this population.

## Trial status

 Protocol version and date - Versions 3, 18 November 2021 

## Data Availability

De-identified participant-level data and statistical code from the trial may be provided by request to researchers who provide a methodologically sound proposal or for participant data meta-analysis at the discretion of the BACK-OFF steering committee. To gain access, data requestors will need to sign a data access agreement.

## Abbreviations

SpA: Juvenile SpA
PRO: Patient-reported outcome
PROMIS: Patient-Reported Outcome Measurement Information System
TNFi: Tumor necrosis factor inhibitor
PCORnet: Patient-Powered Research Network
PRINTO: Pediatric Rheumatology International Trials Organization
DMARD: Disease-modifying antirheumatic drug
SD: Standard deviation
HER: Electronic health record
REDCap: Research Electronic Data Capture
RCI: Reliable change index
SEM: Standard Error of Measurement
IRT: Item response theory
EAP: Expected a posteriori
DSMB: Data and Safety Monitoring Board
ITT: Intention-to-treat
HR: Hazard ratio
K-M: Kaplan-Meier
CTCAE: Common Terminology Criteria for Adverse Events v5.0
